# Navigating uncharted waters: assessing the impact of the COVID-19 pandemic on hematopoietic stem cell transplantation: challenges and innovations

**DOI:** 10.1097/MS9.0000000000002442

**Published:** 2024-08-07

**Authors:** Zaheer Qureshi, Faryal Altaf, Abdur Jamil, Rimsha Siddique, Shivendra Shah

**Affiliations:** a The Frank H. Netter M.D. School of Medicine at Quinnipiac University, Bridgeport, CT; b Department of Internal Medicine, Icahn School of Medicine at Mount Sinai/BronxCare Health System, New York; c Department of Medicine, Samaritan Medical Centre; d Independent Research Associate, Watertown, NY, USA; e Nepalgunj Medical College, Chisapani, Nepal

**Keywords:** challenges, clinical guidelines, COVID-19 pandemic, healthcare adaptations, healthcare resilience, hematopoietic stem cell transplantation, innovations, patient outcomes, risk mitigation, treatment modifications

## Abstract

The COVID-19 pandemic has significantly impacted hematopoietic stem cell transplantation (HSCT), necessitating adaptations across pre-transplant, transplantation, and post-transplant phases. HSCT recipients with compromised immune systems face heightened risks of severe COVID-19 outcomes, including increased mortality. The pandemic prompted significant changes in treatment strategies, with many patients experiencing delays or deferrals in autologous stem cell transplantation (ASCT), alongside adjustments to chemotherapy regimens to prevent disease recurrence. Clinical practices have evolved to address pandemic-related challenges, including a decrease in allo-HSCT procedures, a shift towards using domestic donors and peripheral blood stem cells over bone marrow grafts, and integration of telemedicine to reduce patient burden. These adaptations aim to balance COVID-19 exposure risks with the need for lifesaving HSCT. Innovations in response to the pandemic include stringent infection control measures, modified conditioning regimens, and revised post-transplant care protocols to mitigate infection risks. The importance of optimizing antiviral treatments, exploring new immunomodulatory interventions, and researching broadly neutralizing antibodies for HSCT recipients has been underscored. Despite the difficulties, the pandemic has catalyzed significant learning and innovation in HSCT practices, emphasizing the need for ongoing adaptation and research to protect this vulnerable patient population.

## Introduction

Highlights

- During the COVID-19 pandemic, the healthcare industry was adversely impacted, affecting HSCT through scarce resources.
- The adverse impact on HSCT necessitated alternative transplantation adaptations to reduce severe COVID-19 risks.
- Cryopreservation during the pandemic mitigated transmission of SARS-CoV-2 via the graft before HSCT.
- By utilizing telemedicine, it was critical in enhancing hematopoietic cell transplantation and treatment by reducing the financial and time burdens.
- Similarly, following the clinical guidelines stipulated by different bodies facilitated adaptation to changes in transplantation procedures and transplant centers.

Stem cells throughout the body play a fundamental role in developing different tissues because of their exceptional ability to continuously regenerate themselves and generate a wide range of specialized cells^1^. The characteristics and capabilities of stem cells differ greatly depending on their anatomical location within the body or the tissue compartment they reside in^1,2^. Hematopoietic stem cells (HSCs) possess two distinct abilities: self-renewal and the capacity to differentiate into several types of fully developed blood cells. Hematopoiesis is a continuous developmental process in which hematopoietic stem cells (HSCs) make a succession of cell destiny choices to produce various types of blood cells^3^.

A complex system maintains equilibrium in the generation and upkeep of fully developed blood cells, and it still needs to be fully comprehended in the network of regulatory processes. The interplay between hematopoietic cells and the bone marrow stroma, mediated by soluble and cell-bound cytokines, is crucial in regulating the differentiation and proliferation of these cells^4^. The most basic hematopoietic stem cells (HSCs) contain markers such as the cell surface antigen CD34, as well as receptors for important hematopoietic growth factors like the kinase domain receptor [KDR (flk-1)], vascular endothelial growth factor, and regulators like c-kit and flt-3^5^. These indicators are crucial for the ex vivo amplification of hematopoietic stem cells (HSCs), allowing for their retrieval for transplantation purposes, either from bone marrow or peripheral blood^5^.

The effective recovery of the hematopoietic system after bone marrow ablation depends significantly on the capacity of stem cells injected intravenously to locate and assimilate into the hematopoietic microenvironments or niches within the recipient’s bone marrow^6^. The process, commonly known as “homing,” is complex and involves the step-by-step activation of a sequence of adhesion molecules^7^. One of the critical factors responsible for drawing stem cells to certain areas is a chemokine called stromal cell-derived factor-1 (SDF-1). SDF-1 is an intense attractant for monocytes, lymphocytes, and CD34+ cells, guiding their movement and settling in the bone marrow^8,9^.

The WHO announced COVID-19 a pandemic in March 2020. COVID-19 is caused by the virus known as SARS-CoV-2. As of September 2020, this disease had resulted in a global death toll of 1 million^10^. Individuals diagnosed with cancer face a significantly increased likelihood of being admitted to the intensive care unit, requiring invasive ventilation, and experiencing mortality due to COVID-19 compared to the general population. This increased risk is at least twice as high^11^. Hematopoietic stem cell transplantation (HSCT) recipients may be more susceptible to infection-related and respiratory problems due to their underdeveloped immune systems or organ damage caused by treatment-related toxicities^12^.

Research by Camilleri *et al.* ^13^ found substantial alterations in the treatment plans of transplant-eligible, newly diagnosed multiple myeloma (MM) patients as a result of the COVID-19 pandemic. More than 75% of these individuals underwent significant alterations to their planned treatment courses. More precisely, 39% of patients experienced delays in undergoing autologous stem cell transplantation (ASCT), while 37% did not undergo ASCT as their initial therapeutic choice. A considerable proportion, precisely 75%, of individuals who encountered delays or deferrals in their ASCT underwent maintenance or holding chemotherapy regimens to reduce the likelihood of MM recurrence^13^.

The pandemic had a significant psychological impact on these patients, as many experienced elevated feelings of fear and anxiety associated explicitly with COVID-19^14^. Their heightened awareness of the elevated susceptibility to infection resulting from their impaired immune system intensified their worry. Although patients comprehended the need for adjustments in their healthcare to guarantee their safety amid the pandemic, they had difficulties due to the emotional repercussions of these modifications, specifically the limitations on social connections, which hurt their mental well-being^15^. Surprisingly, despite increased concerns about the possibility of contracting COVID-19, certain patients were still eager to undergo ASCT during this time^13^. Despite the ongoing epidemic, this highlights their resolve to pursue a potentially curative treatment.

Autologous, syngeneic, and allogeneic HSCT play essential roles in aiding hematopoietic recovery after intense chemoradiotherapy, not only for cancerous blood diseases but also for non-blood-related conditions^1,3^. Syngeneic or allogeneic HSCTs play a crucial role in treating acquired illnesses that affect the functioning of the bone marrow, such as aplastic anemia. They also cure congenital deficiencies in blood cell production or immune system function, such as thalassemia and severe combined immunodeficiency syndrome^6,16^. The pandemic’s effect on these treatments underscores the intricate difficulties patients and healthcare personnel encounter in managing the trade-off between the dangers of COVID-19 exposure and the imperative of carrying out lifesaving HSCT procedures^13^.

## Aim of the study

The primary objective of this review is to meticulously examine the multifaceted impact of the COVID-19 pandemic on the entire continuum of HSCT, encompassing pre-transplant considerations, the transplantation process itself, and the extensive period of post-transplant care and follow-up within the context of the pandemic. This includes an in-depth analysis of how the pandemic has influenced patient selection, donor availability, and the logistical aspects of stem cell collection and transplantation. Additionally, the review aims to explore adjusting to conditioning regimens, implementing infection control measures to protect recipients and healthcare providers, and adapting post-transplant care protocols to mitigate COVID-19 risks.

## Impact on HSCT recipients

### Infection risks and outcomes

During the early COVID-19 pandemic, investigations focused on patients who underwent allogeneic hematopoietic stem cell transplantation (allo-HSCT)^17–20^. These studies consistently found a specific set of symptoms in patients who were diagnosed with SARS-CoV-2 infection. The most common symptoms described were fever (65–75%), cough (55–65%), upper respiratory symptoms (20–45%), and asthenia involving 10–49% of patients. Other symptoms seen included a flu-like condition, myalgia, digestive issues, and particular neurological symptoms such as anosmia and dysgeusia. Remarkably, more than 10% of patients were found to be asymptomatic at the time of their COVID-19 diagnosis.

Regarding the necessity for medical intervention, around 32–52% of the patients required supplementary oxygen therapy to control their symptoms effectively^17,21^. The extensive study carried out by the European Society for Blood and Marrow Transplantation (EBMT), involving 382 patients, found no noticeable variation in the clinical manifestation of COVID-19 between individuals who received allogeneic and autologous transplants. This indicates a comparable set of symptoms among different types of transplant recipients^17^.

Piñana *et al.* ^19^ conducted a comparison analysis to assess the clinical manifestations of patients who underwent allo-HSCT and those who did not undergo transplantation but were diagnosed with COVID-19 and had hematologic malignancies. The study revealed no notable disparities in symptoms between the two groups since more than 90% of participants in both cohorts exhibited symptoms. The prevalent symptoms seen were fever, fatigue, and cough. The reason for this closeness in clinical presentation can be linked to patients in these categories sharing an immunosuppressed condition^19^.

The remission of COVID-19 symptoms in recipients of allogeneic hematopoietic stem cell transplantation (allo-HSCT) usually took between 14 and 26 days on average^17,19,21^. Nevertheless, the fact that SARS-CoV-2 RNA remains detectable by positive PCR testing in 5.6% of patients in the EBMT research highlights the difficulty of effectively controlling prolonged viral shedding in this group of individuals with weakened immune systems^17^. An additional concern is establishing the protracted COVID-19 syndrome, characterized by the persistence of symptoms or the occurrence of new issues beyond 4 weeks from the initial acute phase^22^. Common symptoms of this condition include tiredness, difficulty breathing, coughing, chest discomfort, and a general decline in overall well-being. Although limited research has been conducted on the occurrence of extended COVID-19 syndrome in individuals with hematological malignancies, more precise information must be given on how common it is and how it affects recipients of allo-HSCT^22^. In Table 1, a summary of the studies regarding the infection and outcome of HSCT is summarized.

**Table 1:** Summary of the infection rates and outcomes in HSCT patients

Study reference	No. participants	Infection rate	Severity	Mortality	Conclusions on risk factors for severe outcomes
Chinese Study^23^	Not specified	Elevated compared to general population	Greater risk of severe disease	Higher mortality among hematologic malignancy patients	Hematological malignancy increases risk for severe COVID-19 outcomes.
Italian Study^16^	536	Not specified	Not specified	Mortality ratio of 41.3	Older age, progressive disease status, specific diagnoses (e.g., AML, aggressive NHL) associated with worse survival.
Indian Study^24^	Not specified	Not specified	Not specified	20% overall mortality, comparable to another study’s 25%	Hematologic malignancy patients significantly at risk of mortality from COVID-19.
Mirgh *et al.* ^25^	Case report	Not applicable	Managed with tocilizumab	Patient recovered	Tocilizumab might be beneficial for post-HSCT patients with COVID-19, considering individual factors.
Rajendra *et al.* ^26^	6 HSCT patients	Not specified	Favorable outcomes with antivirals and tocilizumab	Not specified	Early intervention with antivirals and tocilizumab could improve outcomes in HSCT patients with COVID-19.
Sharma *et al.* ^21^	Allo-HSCT: 184	Not specified	Short-term follow-up reported	Median follow-up of survivors: 21 days (allo-HSCT)	Short follow-up period post-COVID-19 in HSCT recipients; specific risk factors for severe outcomes not identified.

allo-HSCT, allogeneic hematopoietic stem cell transplantation; AML, acute myeloid leukemia; NHL, Non-Hodgkin's lymphoma.

## Vaccination responses in HSCT patient

Studies on the immune system’s reaction to SARS-CoV-2, ranging from initial vaccine creation to immunity after infection, have revealed that the virus prompts the production of neutralizing antibodies that specifically target different parts of the virus, including the spike (S) protein, its receptor-binding domain (RBD), and the nucleocapsid (N) protein^27^. This last characteristic differentiates between immunity acquired by infection and immunity obtained through vaccination. At first, infection creates a shell of protection for the immune system^28^. However, this defense weakens as time passes, leading to notable decreases in antibody and cellular immunity one year after the infection^29^. Vaccination can enhance or maintain this immunity, as indicated by elevated levels of antibodies, highlighting the significance of vaccines even for individuals who have been previously infected^28,29^. Dimeglio and colleagues have discovered that individuals who have been previously infected may have a more enduring immune response after vaccination, resulting in longer-lasting immunity than those who have not had a prior infection^30^. The prolonged protection could be attributed to a more robust cellular response, increased efficacy of antibodies, or more excellent immunological memory resulting from the viral infection^30^.

Despite new variations, vaccination remained the principal method of preventing SARS-CoV-2 in individuals with HSCT. It was essential to comprehend the factors determining the immune response in high-risk groups, specifically in patients undergoing allogeneic HSCT^31^. Many studies commonly involve the use of decreased-intensity conditioning and unrelated donors. Many patients in these studies were under immunosuppression at the time of vaccination. Additionally, a significant fraction of these patients had a history of graft-versus-host disease (GVHD), and ~20–25% of them have chronic GVHD. The period between transplantation and immunization varies significantly in different studies, focusing on not vaccinating recent transplant recipients immediately. mRNA vaccines were commonly used^32–35^.

Although HSCT patients have a reduced response rate compared to healthy controls, most of them, 60%, showed serological positivity after vaccination. Additionally, a large number of HSCT patients reached antibody levels that are deemed protective. Notably, T-cell depletion techniques such as ATG have demonstrated varied effects on vaccination response, emphasizing the intricate relationship between transplant conditioning and immunological reactivity to vaccines^36^. Factors identified as harmful to the response of vaccines include recent transplantation, continuous immunosuppression, and therapies that alter the process of immune reconstitution, such as rituximab^36^.

An extra dose of the vaccine is indicated for patients who have had allo-HSCT and had unsatisfactory initial responses to enhance their humoral response^31^. This approach has shown encouraging rates of seroconversion. Low B cell numbers and recent immunosuppressive therapies are identified as negative indicators^37^. Significantly, additional doses have substantially increased the levels of neutralizing antibodies against other strains of SARS-CoV-2, such as Delta and Omicron. This highlights the advantage of booster doses in this group^31,38^.

Research monitoring the longevity of antibodies following vaccination showed a decrease after six months, yet a significant number of individuals still maintained levels that provide protection. Recent administration of rituximab, systemic immunosuppression, and low lymphocyte counts at the time of immunization were associated with reduced antibody levels at 6 months. Although there has been a decrease, a solid and high level of antibodies after vaccination is associated with long-lasting protection, indicating that a solid initial immune response may help prevent quick loss of immunity^31,36–38^.

Allo-HSCT recipients often exhibit favorable tolerance to SARS-CoV-2 vaccinations, with predominantly mild to moderate side effects^39^. In multiple investigations, more than 50% of the patients experienced adverse effects, with occurrence rates ranging from 48 to 80%. The predominant adverse effects seen were localized reactions at the site of vaccination administration, affecting 30–86% of patients^40,41^. These reactions encompassed symptoms such as pain, redness, or swelling. Aside from local reactions, patients also had systemic symptoms, such as asthenia in 20–41% of cases, myalgia and headache in 15–30% of cases, and chills in 7–15% of instances^42^. Table 2 summarizes an Overview of the immune response to vaccination against SARS-CoV-2 in HSCT and CAR-T-cell therapy recipients^43^.

**Table 2:** Immune response to vaccination against SARS-CoV-2 in HSCT and CAR-T-cell in HSCT

Study (Wu *et al*., 2022)^43^	Details
Total no. studies included	46
Total no. participants	4757 HSCT and 174 CAR-T recipients
Vaccine type	Mainly mRNA vaccines (BNT162b2, mRNA-1273)
Number of doses analyzed	Varied across studies; responses after partial (1 dose), complete (2 doses), and booster (3rd dose) vaccination assessed
Time post-transplant	Ranged from <6 to ≥12 months
Observed efficacy (antibody response rates)	HSCT: 81.4% after complete vaccination; 40.8% after 1 dose; 78.6% after 3rd dose - CAR-T: Combined serological response rate of 35.9%
Noted side effects	Mild to moderate; predominantly local reactions (pain, redness, swelling), systemic symptoms (asthenia, myalgia, headache, chills)
Significant findings	Time interval from transplant to vaccination significantly affects humoral response in HSCT recipients - Seroconversion higher in BCMA-based CAR-T than CD19-based CAR-T recipients - IST and lymphopenia at vaccination time associated with seronegative response in HSCT recipients
Recommendations	Adapted vaccination strategy may be required for HSCT and CAR-T recipients, with further research on booster dose efficacy

HSCT, hematopoietic stem cell transplantation.

## Changes in clinical practices

The SARS-CoV-2 pandemic had a global influence, significantly impacting hospital organizations. This necessitated several adjustments in daily medical procedures^44^. During this crisis, there was a decline in the number of allo-HSCT procedures recorded in many countries^45^, as indicated in the EBMT report^46^. The reduction amounted to 5.1% in 2020 compared to 2019. These differences coincide with the EBMT guidelines, which suggest delaying transplantation for chronic non-malignant disease if feasible^45^. The EBMT report and other studies have shown that the decrease primarily affects non-malignant diseases such as sickle cell disease (decreased by −30.9%) and thalassemia (decreased by −19.6%)^46^. Hematological malignancies, on the other hand, only experience a slight decrease, with acute myeloid leukemia decreasing by −2.1%, myelodysplastic syndromes decreasing by −4.3%, myeloproliferative syndromes decreasing by −1.2%, and acute lymphoblastic leukemia decreasing by −0.7%. In all of these investigations, there was also a drop in the transplantation of lymphoid diseases. However, this decline may be attributed to the introduction of new medicines, such as chimeric antigen receptor (CAR)-T-cell therapies, rather than being caused by the COVID-19 pandemic. Furthermore, there were no discernible disparities in age, sex, or comorbidity index between patients who underwent transplantation during the COVID-19 era and those who underwent transplantation before the pandemic^45,47^. This indicates that COVID-19 did not lead to the implementation of more rigorous eligibility criteria for allogeneic transplantation.

The selection of donors has also been affected by the COVID-19 pandemic. During the crisis, international bone marrow donor centers saw disruptions in foreign traffic, potentially leading to higher utilization of domestic donors. The Deutsche Knochenmarkspenderdatei (DKMS) report showed a 37% usage of domestic donors compared to 22% previously, making it more convenient to handle^44^. Although the United States study did not find any difference^47^, the two European trials showed a higher occurrence of haplo-identical transplants (an increase from 6.2 to 12%)^46^. Similar findings were found in Chinese studies^48,49^. While utilizing a family donor may offer greater ease of management and a higher likelihood of successful stem cell collection, the increase in utilization can also be attributed to improved understanding of haplo-identical transplantation and enhanced prevention of GVHD through the use of post-transplant cyclophosphamide (PTCy).

**Figure 1:**
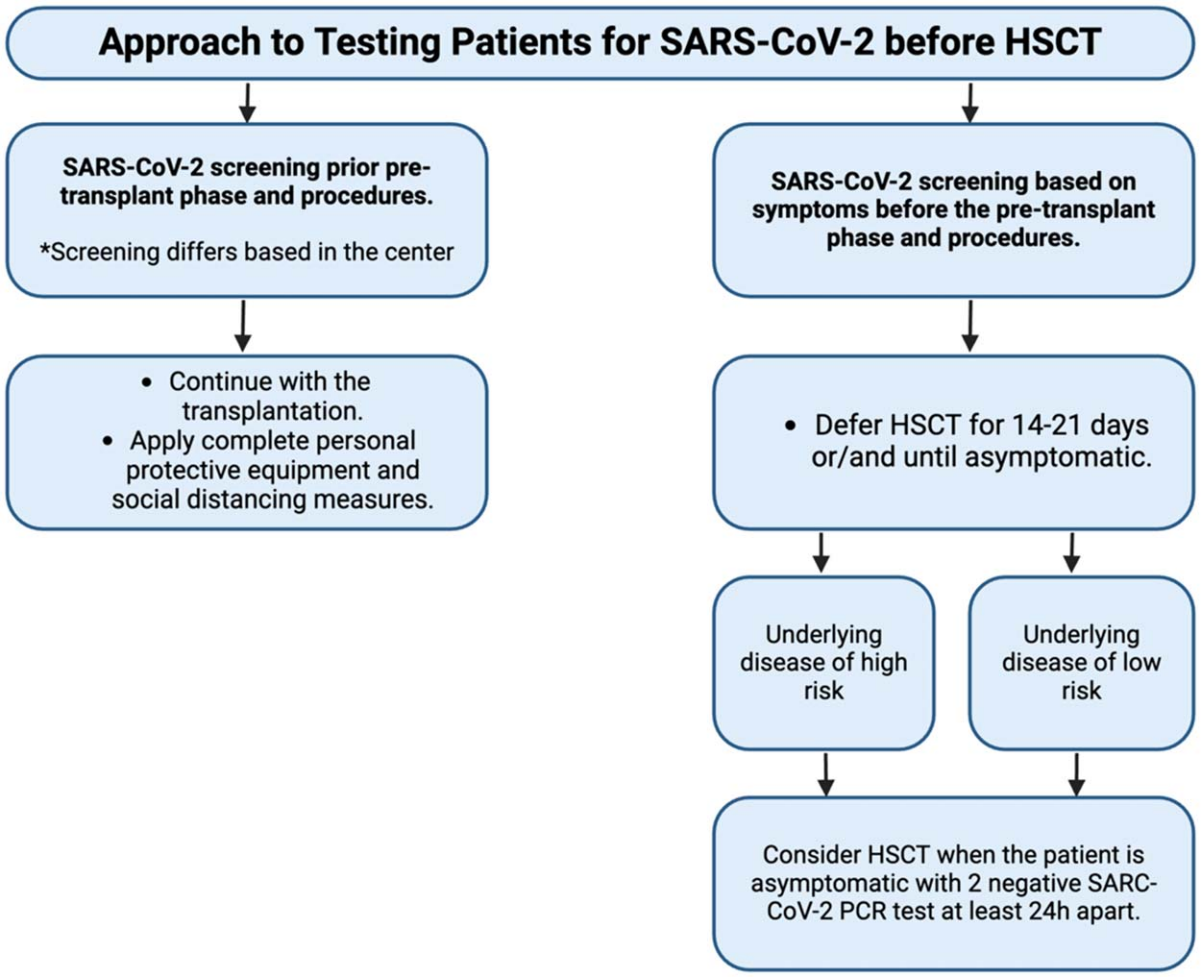
Approach to testing patients for SARS-COV-2 before HSCT. HSCT, hematopoietic stem cell transplantation.

The COVID-19 pandemic has led to a significant decline in bone marrow (BM) graft utilization compared to peripheral blood stem cells (PBSC). According to the EBMT study, there has been a 37% decrease in the use of BM grafts^46^. The preference for PBSC transplants can be attributed to the temporary shutdown of operating rooms during the pandemic, making collecting bone marrow more challenging. Furthermore, the guidelines established by EBMT and the American Society for Transplantation and Cellular Therapy (ASTCT) during the crisis advocated for cryopreservation, which rapidly became the accepted norm^50^. The more significant technical challenge of freezing and thawing allogeneic bone marrow cells, compared to mobilized PBSC, could account for the increased utilization of PBSC^50^. The introduction of cryopreservation aimed to mitigate two significant hazards. The first issue is the failure to collect a graft due to either a symptomatic donor infected with SARS-CoV-2 or a travel incident. This can result in a lack of available graft for a patient who has already begun receiving the pre-transplant conditioning regimen. The second concern is the potential transmission of SARS-CoV-2 through the graft. This can be avoided by postponing the transplantation and monitoring the donor’s status for a few days after collection^50^—the approach to testing patients for SARS-CoV-2 before HSCT is shown in Figure 1. Clinical trials and observational studies on the impact of the COVID-19 pandemic on HSCT are shown in Table 3. Key findings from the clinical trials and observational studies on the impact of COVID-19 on HSCT recipients are shown in Table 4.

**Table 3:** Clinical trials and observational studies on the impact of the COVID-19 pandemic on hematopoietic stem cell transplantation (HSCT)

Study title	Type	Objective	Key metrics	Findings	Location
Clinical Trials and Observational Studies on the Impact of the COVID-19 Pandemic on Hematopoietic Stem Cell Transplantation (HSCT)	Retrospective Observational Cohort Study	Describe the impact of COVID-19 in patients treated with autologous stem cell transplantation (ASCT)	Overall survival, COVID-19-related mortality, time of infection relative to ASCT, hospitalization duration, oxygen therapy requirements	Higher mortality in ASCT patients with COVID-19; significant need for intensive care	Sweden
Observational Cohort Study by CIBMTR	Observational cohort study	Describe characteristics, treatment patterns, and factors associated with outcomes of HSCT recipients with COVID-19	Mortality rates, infection timing, demographic factors, underlying disease	Higher mortality in HSCT patients with COVID-19, especially in those over 50, males, and those infected within 12 months of transplantation; lymphoma indicated higher risk	Multiple Countries
Impact of COVID-19 Vaccine on Allogeneic Hematopoietic Stem Cell Transplantation	Observational cohort study	Evaluate the impact of COVID-19 vaccination on safety and efficacy of allo-HSCT recipients	Survival rates, infection rates, immune response	Mixed results on efficacy, but highlighted importance of vaccination in reducing severe outcomes	China
Effectiveness of COVID-19 Vaccine in HSCT Patients	Interventional clinical trial	Assess the immunogenicity and effectiveness of the Pasocovac vaccine in HSCT patients	Antibody titers, adverse reactions	Significant immune response post-vaccination, with manageable adverse reactions	Iran
Outcome of COVID-19 in Allogeneic Stem Cell Transplant Recipients (EPICOVIDEHA)	Retrospective multicenter study	Analyze outcomes and risk factors for mortality in allogeneic HSCT patients with COVID-19	Severity of infection, mortality rates, risk factors	High mortality, particularly in older patients, those with comorbidities, and recent transplant recipients	Europe

**Table 4:** Key findings from the clinical trials and observational studies on the impact of COVID-19 on HSCT recipients

Finding	Details
Mortality rate	Pooled prevalence of death among HSCT recipients with COVID-19: 17%
Risk factors for higher mortality in allogeneic HSCT	Older age
	Male sex
	Developing COVID-19 within 12 months post-transplant
	Lymphoma as disease indication
Risk factor for higher mortality in autologous HSCT	Low absolute lymphocyte count (<0.3×10^9^/l)
Severe/critical COVID-19	Pooled prevalence of severe/critical COVID-19 among HSCT recipients: 24%
Timing of COVID-19	Most HSCT recipients who developed COVID-19 were >1-year post-transplant and not receiving immunosuppression
Factors not associated with increased mortality	Race
	Ethnicity
	Comorbidities at transplant
	Recent immunosuppression

HSCT, hematopoietic stem cell transplantation.

## Ethical and logistical challenges

Healthcare institutions faced substantial constraints in terms of resources during the pandemic. Although there were no extensive shortages of essential resources, public hospitals encountered more challenges in distributing resources than the private sector due to higher patient volumes^51^. The availability of beds for HSCT patients was diminished, especially in the early phases of the pandemic, when the transplant wards were converted into COVID-19 isolation units^50^. Ensuring the security of a solitary room with adequate ventilation and air filters for every patient posed significant logistical difficulties^52^. The redirection of medical staff to care for COVID-19 patients resulted in labor shortages, increasing the burden per person for transplant teams. In pediatric care, the challenges of allocating resources were less significant due to the fewer patients undergoing HSCT compared to adults. These constraints led to delays and required limitations on the quantity of transplants carried out^51,52^.

Delaying or deferring HSCT during the COVID-19 pandemic posed significant ethical considerations and complex decision-making processes. The key factors included: Weighing Risks and Benefits Clinicians had to carefully weigh the risks of delaying HSCT, which could lead to disease progression and poorer outcomes, against the dangers of proceeding with HSCT during the pandemic, which increased exposure to COVID-19 and potential complications^50^. Patient Characteristics and Disease Severity The decision to delay or proceed with HSCT was highly individualized, considering factors such as patient age, comorbidities, disease stage, and risk stratification. Despite the pandemic risks, more aggressive diseases or advanced stages often necessitated prompt HSCT^52^. Resource Allocation and Access Limited healthcare resources and access to transplant centers during the pandemic further complicated decision-making, requiring prioritization of cases and potential delays for some patients. Informed Consent and Patient Autonomy Ensuring patients and families were fully informed about the risks and benefits of delaying or proceeding with HSCT during the pandemic was crucial for respecting patient autonomy and shared decision-making. Overall, the ethical considerations and decision-making processes during the COVID-19 pandemic required a delicate balance between disease-related factors, pandemic risks, resource availability, and patient preferences, underscoring the importance of individualized care and open communication^50,52^.

## Innovations and lessons learned

Telemedicine has become essential in hematopoietic cell transplantation (HCT) and cellular treatment. It can improve access to specialized care and reduce in-person consultations’ time and financial burdens. A study conducted in 2015 examined the availability of HCT services in the United States (US). The study found that 229 facilities offered these services, catering to a population of around 306 million. This translates to an average of one HCT facility for every 1.3 million people in the US^53^. Approximately 46% of the population in the United States lived within a 30-minute distance from an HCT center that is suitable for their age. However, individuals outside this area faced restricted access to HCT care. This implies that a significant portion, ranging from 30 to 50% of the United States population, would need to travel 120–180 min to access healthcare services at an HCT center. A study conducted at an alloHCT center in Brazil found that most patients (*n*=232) had cell phones or personal computers, which allowed them to take part in a survey assessing the time and cost difficulties associated with in-person clinic visits^54^. The findings indicated that 33% of patients had a journey time of more than 120 min to and from the clinic, while 42% had commuting expenses surpassing USD 10.00. In addition, 38% of the participants reported experiencing varying levels of physical weakness or disability, while 28% expressed dissatisfaction with the extended waiting periods for in-person appointments with their doctor. Regrettably, the travel duration, the waiting time at the office, and the time spent during the office visit result in both temporal and financial burdens for patients. Consequently, patients may encounter difficulties in complying with subsequent sessions. Telemedicine enhances the availability of HCT treatments and minimizes the adverse effects of time and financial burdens, especially for patients deemed medically stable following HCT^54^.

## Effectiveness of different approaches

In one case, a 7-year-old pediatric patient with acute lymphoblastic leukemia faced significant delays in receiving a hematopoietic stem cell transplant due to travel restrictions affecting donor availability. The medical team had to use cryopreserved stem cells from a less optimal alternative donor. Although the transplant was ultimately successful, the patient experienced an increased incidence of GVHD, illustrating the critical impact of pandemic-related delays on donor selection and patient outcomes^55^. Another case involved a 55-year-old adult patient with multiple myeloma who required urgent transplantation. Due to hospital capacity limitations and stringent infection control measures, the patient’s management was modified to include more outpatient care and telehealth follow-ups. This adaptation allowed the patient to undergo transplantation without contracting COVID-19. Still, it led to a prolonged hospital stay due to complications, highlighting the balance between maintaining patient safety and managing resource constraints during the pandemic^56^. A third case featured a 32-year-old patient with relapsed Hodgkin lymphoma undergoing a second transplant. This patient struggled with limited access to supportive care services and psychosocial support due to the pandemic. The introduction of virtual support groups and increased mental health services via telemedicine improved the patient’s psychological well-being, though physical recovery was delayed. This case underscores the importance of virtual support systems in addressing the psychological impacts of prolonged isolation during the pandemic^57^.

A retrospective study in Brazil found that among 49 hospitalized HSCT recipients with COVID-19, the all-cause mortality rate was 40.8%. Mechanical ventilation and chest CT involvement more than or equal to 50% at diagnosis were associated with higher mortality^55^. A multicenter study across ten centers in the Middle East reported on 91 HSCT recipients with COVID-19. The mortality rate was 4.4%, with 53% requiring hospitalization and 14% requiring ICU admission. Time from transplant more than 6 months was associated with lower admission rates and severity^58^. A study of 492 allogeneic HSCT recipients with COVID-19 during the Omicron wave found a 15.7% incidence of moderate-to-severe COVID-19. Risk factors included corticosteroid use within three months before diagnosis and time from transplant less than 6 months^59^. In the Brazilian study, non-vaccinated HSCT recipients had significantly higher mortality (51.4%) compared to vaccinated recipients (14.2%)^55^. The Middle East study found antibody responses in 67% of evaluable HSCT recipients after COVID-19 infection^56^. The survey of Omicron cases showed corticosteroid use within three months before COVID-19 diagnosis (OR 2.129) and time from transplant less than 6 months (OR 2.667) were risk factors for moderate-to-severe disease^6^. While the studies varied in outcomes, they consistently found higher risks of severe COVID-19 and mortality in HSCT recipients, especially within the first 6–12 months post-transplant and in unvaccinated patients.

## Cellular senescence and inflammaging

Cellular senescence and inflammaging cellular senescence and inflammaging are closely interconnected processes contributing to age-related diseases and declining tissue function^60^. Mesenchymal stromal cells (MSCs) play a crucial role in these processes, as their secretome can drive inflammaging when the cells become senescent. External stressors like skin, the body’s largest organ, are continuously exposed to external stressors such as UV radiation, air particulate matter, and the human microbiome. These factors can contribute to cellular senescence and inflammaging in the skin microenvironment. Infectious diseases, while not explicitly mentioned in the search results, it’s well-established that contagious diseases can act as external stressors that potentially accelerate cellular senescence and inflammaging^60^. This is particularly relevant in COVID-19, associated with increased inflammation and potential long-term effects on cellular aging.

Cellular senescence and inflammaging could impact the success of hematopoietic stem cell transplantation^60,61^. Senescent cells in the bone marrow microenvironment might affect the engraftment and function of transplanted stem cells. Additionally, the pro-inflammatory state associated with inflammaging could influence immune reconstitution post-transplantation^61^. The relationship between cellular senescence, inflammaging, and COVID-19 is complex. Pre-existing inflammaging in older individuals may contribute to more severe COVID-19 outcomes. Conversely, SARS-CoV-2 infection might accelerate cellular senescence and exacerbate inflammaging, potentially contributing to long-term complications.

## Conclusion

The COVID-19 pandemic has substantially impacted illness and death rates in individuals who have undergone HSCT. The increased susceptibility emphasizes the need for focused research endeavors. It is crucial to prioritize optimizing antiviral treatments, investigating new immunomodulatory interventions, and the exploration of broadly neutralizing antibodies specifically for patients undergoing HSCT.

## Ethical approval

Our study was a narrative review and therefore, did not involve patients. Thus, ethical approval from the ethics committee was not applicable.

## Consent

Our study was a narrative review (a secondary study). As a result, the study did not involve patients; hence, consent was not applicable.

## Source of funding

Not applicable.

## Author contribution

Z.Q.: conceptualization; writing—original draft preparation; supervision. F.A.: visualization; writing—original draft preparation. A.J.: writing—reviewing and editing. R.S.: writing—manuscript writing and editing. S.S.: writing—editing and reviewing.

## Conflicts of interest disclosure

The authors declare no conflict of interest.

## Research registration unique identifying number (UIN)

Not applicable.

## Guarantor

Zaheer Qureshi.

## Data availability statement

Not applicable.

## Provenance and peer review

Our paper was not invited.
